# Influence of tissue context on gene prioritization for predicted transcriptome-wide association studies

**Published:** 2019

**Authors:** Binglan Li, Yogasudha Veturi, Yuki Bradford, Shefali S. Verma, Anurag Verma, Anastasia M. Lucas, David W. Haas, Marylyn D. Ritchie

**Affiliations:** Genomics and Computational Biology Program, University of Pennsylvania Philadelphia, PA 19104, USA; Department of Genetics, University of Pennsylvania Philadelphia, PA 19104, USA; Department of Genetics, University of Pennsylvania Philadelphia, PA 19104, USA; Department of Genetics, University of Pennsylvania Philadelphia, PA 19104, USA; Department of Genetics, University of Pennsylvania Philadelphia, PA 19104, USA; Department of Genetics, University of Pennsylvania Philadelphia, PA 19104, USA; Departments of Medicine, Pharmacology, Pathology, Microbiology & Immunology, Vanderbilt University School of Medicine, Nashville, TN, and Department of Internal Medicine, Meharry Medical College, Nashville, TN, USA; Department of Genetics, Institute for Biomedical Informatics, University of Pennsylvania Philadelphia, PA 19104, USA

**Keywords:** TWAS, integrative, context, PrediXcan, UTMOST

## Abstract

Transcriptome-wide association studies (TWAS) have recently gained great attention due to their ability to prioritize complex trait-associated genes and promote potential therapeutics development for complex human diseases. TWAS integrates genotypic data with expression quantitative trait loci (eQTLs) to predict genetically regulated gene expression components and associates predictions with a trait of interest. As such, TWAS can prioritize genes whose differential expressions contribute to the trait of interest and provide mechanistic explanation of complex trait(s). Tissue-specific eQTL information grants TWAS the ability to perform association analysis on tissues whose gene expression profiles are otherwise hard to obtain, such as liver and heart. However, as eQTLs are tissue context-dependent, whether and how the tissue-specificity of eQTLs influences TWAS gene prioritization has not been fully investigated. In this study, we addressed this question by adopting two distinct TWAS methods, PrediXcan and UTMOST, which assume single tissue and integrative tissue effects of eQTLs, respectively. Thirty-eight baseline laboratory traits in 4,360 antiretroviral treatment-naïve individuals from the AIDS Clinical Trials Group (ACTG) studies comprised the input dataset for TWAS. We performed TWAS in a tissue-specific manner and obtained a total of 430 significant gene-trait associations (q-value < 0.05) across multiple tissues. Single tissue-based analysis by PrediXcan contributed 116 of the 430 associations including 64 unique gene-trait pairs in 28 tissues. Integrative tissue-based analysis by UTMOST found the other 314 significant associations that include 50 unique gene-trait pairs across all 44 tissues. Both analyses were able to replicate some associations identified in past variant-based genome-wide association studies (GWAS), such as high-density lipoprotein (HDL) and *CETP* (PrediXcan, q-value = 3.2e-16). Both analyses also identified novel associations. Moreover, single tissue-based and integrative tissue-based analysis shared 11 of 103 unique gene-trait pairs, for example, *PSRC1*-low-density lipoprotein (PrediXcan’s lowest q-value = 8.5e-06; UTMOST’s lowest q-value = 1.8e-05). This study suggests that single tissue-based analysis may have performed better at discovering gene-trait associations when combining results from all tissues. Integrative tissue-based analysis was better at prioritizing genes in multiple tissues and in trait-related tissue. Additional exploration is needed to confirm this conclusion. Finally, although single tissue-based and integrative tissue-based analysis shared significant novel discoveries, tissue context-dependency of eQTLs impacted TWAS gene prioritization. This study provides preliminary data to support continued work on tissue context-dependency of eQTL studies and TWAS.

## 1. Introduction

Improving antiretroviral therapy (ART) efficacy and safety is an ongoing goal for addressing the HIV pandemic. According to the Joint United Nations Programme on HIV and AIDS (UNAIDS) (http://aidsinfo.unaids.org/), approximately 36.7 million people worldwide were living with human immunodeficiency virus (HIV) in 2016. Over the past three decades there has been immense progress on HIV care and treatment, and in 2017 there were about 20.9 million HIV-positive people who had access to ART. The connection of genomics with pharmacology has led to the discovery of numerous single nucleotide polymorphisms (SNPs) in drug absorption, distribution, metabolism, and elimination (ADME) genes and off-target genes. Many SNPs have been related to effects and/or pharmacokinetics of antiretroviral drugs^1–6^. However, most trait-related SNPs lack connections to actual functional genes, which suggests the need for alternative analysis approaches.

The emerging field of transcriptome-wide association studies (TWAS) offer a new way to directly identify gene-trait associations via integration of genotypic data and expression quantitative trait loci (eQTLs). eQTLs are an important class of genetic functional elements, which affect transcriptional regulation on target genes. Integration of eQTL information with genotypic data allows TWAS to estimate the extent to which a gene’s expression level is regulated by genetic variants and how this correlates with traits of interest^8^. The Genotype Tissue Expression Project (GTEx^7^) provides the data and the opportunity to identify eQTLs and estimate effect sizes for multiple human tissues (44 tissues in GTEx v6p). With GTEx, TWAS can explore gene-trait associations on tissues whose gene expression profiles are otherwise hard to obtain, such as liver and heart. However, current TWAS focuses primarily on eQTLs identified in a tissue-by-tissue manner, while many studies have either acknowledged or supported the power of an integrative tissue context in identifying single-tissue and multi-tissue eQTLs^9,10^.

**Figure 1.**
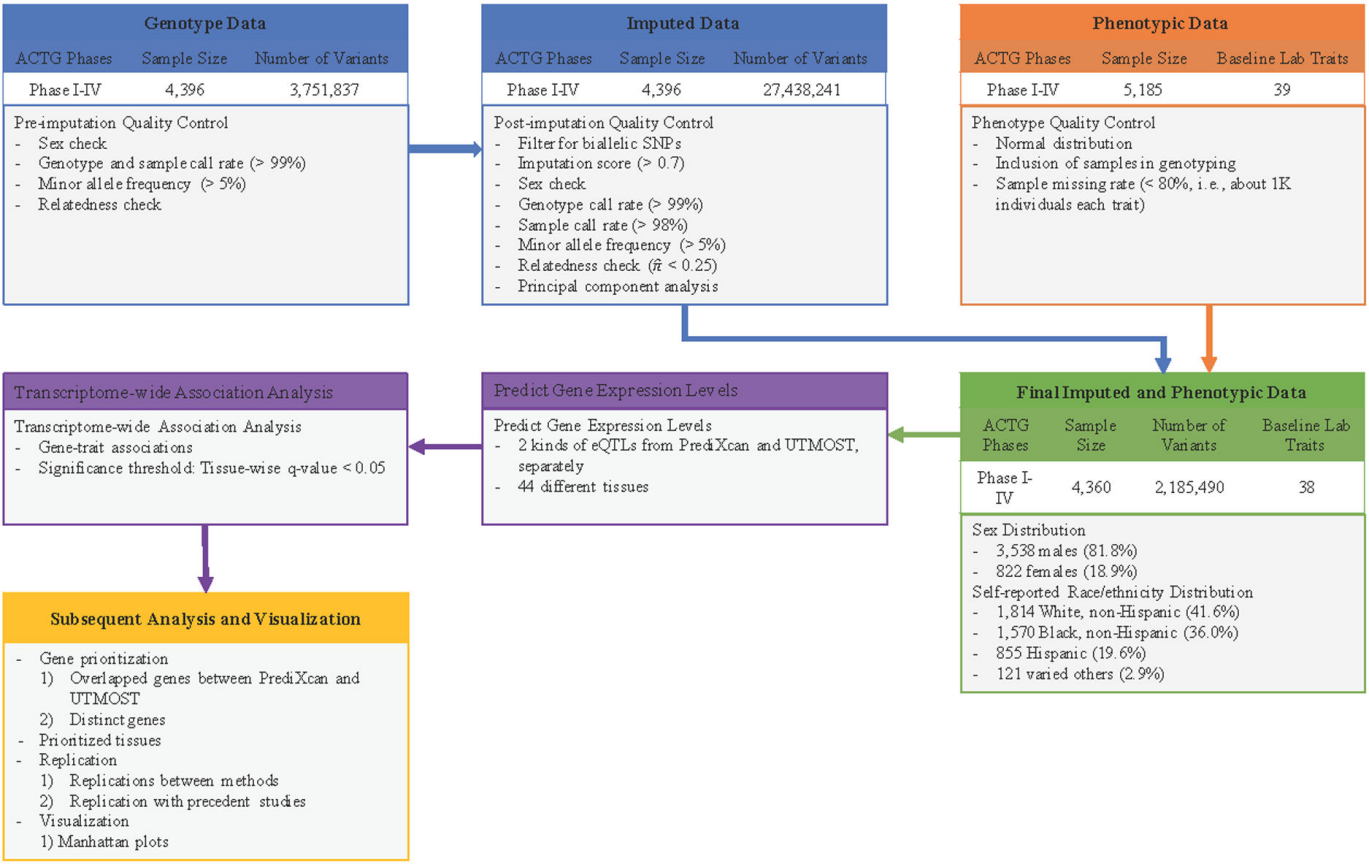
This study investigates the influence of tissue context-dependency of eQTLs on TWAS gene prioritization by comparing two distinct TWAS methods, PrediXcan and UTMOST. PrediXcan assumes single tissue context of eQTLs, while UTMOST assumes eQTLs to possibly have effects in multiple tissues.

In this study, we aimed to address whether and how single tissue and integrative tissue context of eQTLs influence TWAS gene prioritization by comparing two distinct TWAS methods, PrediXcan^11^ and Unified Test for MOlecular SignaTures (UTMOST^12^). PrediXcan uses elastic-net regression model and identifies eQTLs in a tissue-by-tissue manner. UTMOST adopts group-lasso and search through all tissues at once to spot eQTLs of a certain gene. This strategy allows UTMOST to identify single-tissue specific eQTLs similar to PrediXcan but increase the chance of detecting multi-tissue eQTLs. Here, 38 baseline (i.e. pre-ART) laboratory values and genotypic data of 4,360 ACTG clinical trials participants from multiple previous studies^13–19^ comprised the input for TWAS. Genotyping had been previously generated in multiple phases with Illumina assays: 650Y (phase I), 1M Duo (phase II and III), or Human Core Exome (phase IV). We performed the two TWAS methods separately in a tissue-specific manner (i.e. 44 tissues) (Figure 1). If tissue context-dependency of eQTLs did not affect TWAS gene prioritization, we expected to observe shared gene-trait associations between single tissue-based analysis (PrediXcan) and integrative tissue-based analysis (UTMOST). The results partially supported this hypothesis, but also suggested varied gene prioritization abilities of single tissue-based and integrative tissue-based approaches respectively. The former found more unique gene-trait pairs, while the latter tended to prioritize genes expressed in multiple tissues. This study provides supportive evidence for tissue context-dependency of eQTLs and its impact on TWAS gene prioritization.

## 2. Methods

### 2.1. Data and Study Participants

In this study, we used four different genotyping phases of ACTG studies in a combined dataset that included samples and data from participants in prospective, randomized ART-naïve treatment trials^13–19^. Clinical trial designs and results, and results of a genome-wide pleiotropic study results for baseline laboratory values have been described elsewhere^13–21^.

### 2.2. Quality Control

#### 2.2.1. Genotypic data

A total of 4,393 individuals were genotyped in four phases. Phase I was genotyped using Illumina 650Y array; Phase II and III were genotyped using Illumina 1M duo array; Phase IV was genotyped using Illumina HumanCoreExome BeadChip.

The computational preparation of genotypic data included pre-imputation quality control (QC), imputation, and post-imputation quality control. Pre- and post-imputation quality control followed the same guidelines^22^ and used PLINK1.90^23^ and R programming language. Imputation was performed on ACTG phase I-IV combined genotype data. Genotyped variants surviving the preimputation quality control comprised the input datasets for imputation, which used IMPUTE2^24^ with 1000 Genomes^25^ Phase 1 v3 as the reference panel. ACTG phase I-IV combined imputed data had 4,941 individuals and 27,438,241 variants. The following procedures/parameters were used in the post-imputation quality control by PLINK1.90: sample inclusion in phase I-IV phenotype collection, biallelic SNP check, imputation score (> 0.7), sex check, genotype call rate (> 99%), sample call rate (> 98%), and minor allele frequency (MAF > 5%), and relatedness check $\left(\hat{\pi }>0.25\right)$. Subsequent principal component analysis (EIGENSOFT^26^) projected remaining individuals onto the 1000 Genomes Project sample space to examine for population stratification. The first three principal components were used as covariates to adjust for population structure in the subsequent analysis. The final QC’ed ACTG phase I-IV combined imputed data contained 2,185,490 genotyped and imputed biallelic SNPs for 4,360 individuals (Figure 1).

#### 2.2.2. Phenotypic data

The ACTG clinical trials included in this analysis collected baseline (i.e., pre-ART) laboratory traits from 5,185 ART-naïve individuals. We only retained individuals who have been genotyped and traits that were normally distributed and met a criterion of phenotype missing rate < 80%. The final combined phenotype dataset of ACTG genotyping phase I-IV retained 38 traits and the same number of individuals as the QC’ed imputed dataset (Figure 1).

### 2.3. Predict Unmeasured Gene Expression Levels

We adopted two TWAS methods, PrediXcan and UTMOST, to predict unmeasured gene expression levels in a tissue-specific manner. PrediXcan and UTMOST have estimated SNP effect sizes on gene expression levels in 44 tissues, which are available at http://predictdb.org/ and https://github.com/Joker-Jerome/UTMOST, respectively. The PrediXcan and UTMOST scripts were pulled from their GitHub project repositories on April 23^rd^ and Jun 6^th^, 2018, respectively.

PrediXcan and UTMOST followed the same multivariate models. Let *N* denote the sample size and *M* denote the number of eQTLs in a certain gene. A gene’s expression level can be predicted using the multivariate model as follows:

(1) E=Xβ

where *E* is the *N ×* 1 vector of predicted gene expression levels of the gene, *X* is the *N × M* matrix of genotypes, and *β* is the *M* × 1 vector of eQTLs’ estimated regulatory effects on the gene.

Predicted gene expression levels were likely to differ between the two methods as each has a different hypothesis of eQTL regulatory mechanisms in terms of tissue context-dependency. To discover trait-related tissues without assumptions, we predicted gene expression levels in 44 tissues.

### 2.4. Transcriptome-wide Association Analysis

We tested for gene-trait associations by performing transcriptome-wide association tests on predicted gene expression levels and ACTG baseline lab traits using PLATO^27,28^. All baseline labtraits included in this study were continuous and thus were modeled using linear regression. Age, sex, and the first three principal components calculated by EIGENSOFT were included as covariates in linear models to adjust for sampling biases and underlying population structure. PrediXcan and UTMOST have different degrees of diversity in the number of eGenes and gene-trait associations among tissues. To avoid biases due to an uneven number of associations among tissues, p-values were adjusted using FDR with using Benjamini–Hochberg procedure^29^ in a tissue-specific manner. For this study, we consider gene-trait associations significant if they had single tissue-wise q-value < 0.05.

## 3. Results

We compared the influence of tissue context-dependency of eQTLs on TWAS gene prioritization by comparing single tissue-based analysis (PrediXcan) and integrative tissue-based analysis (UTMOST). We performed TWAS on ACTG phase I-IV combined datasets. The data aggregation of ACTG phase I-IV provided a larger sample size to ensure the power of identifying gene-trait association. QC procedures left the ACTG phase I-IV combined imputed data with 4,360 individuals and 2,185,490 SNPs. There were 38 baseline lab traits in the final phenotypic datasets.

Single tissue-based and integrative tissue-based analysis identified a total of 430 significant gene-trait associations (103 unique gene-trait pairs regardless of tissue, q-value < 0.05) and share 11 unique gene-trait pairs. Single tissue-based analysis identified 116 of the 430 significant associations (64 unique gene-trait pairs), encompassing 41 genes, 17 traits, and 28 tissues. Integrative tissue-based analysis identified the remaining 314 significant associations (50 unique gene trait pairs), encompassing 38 genes, 20 traits, and all 44 tissues.

### 3.1. Tissue Context-dependency Influenced TWAS Gene Prioritization

Gene prioritization results from single tissue-based analysis (PrediXcan) and integrative tissue-based analysis (UTMOST) were compared to evaluate the influence of tissue context-dependency of eQTLs on TWAS. Single and integrative tissue-based analyses shared 11 of 103 unique gene-trait pairs regardless of tissue (Table 1). Several of these replicated the findings of previous studies (Table 2). The lowest p-value by integrative tissue-based analysis was for *MROH2A*-total bilirubin levels^20^ (UTMOST, q-value = 6.0e-27), which had a moderate p-value from single tissue-based analysis (q-value = 0.005). Another replication was between *PSRC1* and two lipid-related traits, cholesterol and LDL, which have been reported in other studies^30–33^. Although it was *SORT1*, which neighbors *PSRC1*, that has been functionally related to LDL via mice knockdown experiments^34^. *ALDH5A1* and *GPLD1* have been associated with the liver function test, alkaline phosphatase (ALP)^35^. In the cases of *PSRC1*, *ALDH5A1*, and *GPLD1*, integrative tissue-based analysis (UTMOST) prioritized the genes in their biological function-related organ, liver, which was not always the case for single tissue-based analysis (PrediXcan). Possible novel associations were observed between absolute neutrophil count and *C1orf204*^36^, *ATF6*, and *VANGL2*^37^.

### 3.2. Single Tissue-based Analysis Found a Greater Number of Unique Gene-trait Associations

**Figure 2.**
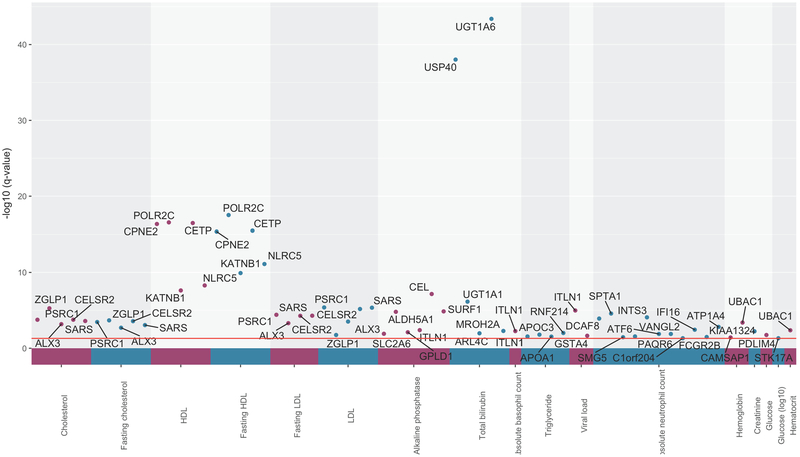
Manhattan plot of gene-trait associations identified by PrediXcan. X-axis showed only significant traits. Y-axis was the q-value transformed by -log10. For simplicity, the plot only shows the lowest p-value of a gene-trait pair, which may appear in multiple tissues.

Single tissue-based analysis using PrediXcan identified 64 unique gene-trait association across different tissues (Figure 2). Some associations have been reported previously (Table 2). PrediXcan associated total bilirubin levels with *UGT1A1*^20^ (skin, q-value = 7.1e-07) and *MROH2A*^20^ (adipose, q-value = 0.005), and LDL and cholesterol to *CELSR2*^30,38,39^ (most significant with LDL in brain, q-value = 6.7e-06). HDL was associated with *CETP*^20,32^ (most significant in colon with q-value = 3.2e-17) and *NLRC5*^38^ (adrenal gland, q-value = 7.8e-12). Triglyceride was associated with *APOA1*^30,39^ (brain, q-value = 0.029) and *APOC3*^30,39^ (heart, q-value = 0.016).

Single tissue-based analysis identified novel gene-trait associations, which warrants further investigation. One interesting example was the association of *ITLN1* with multiple traits, including HIV-1 viral load, triglyceride, and total neutrophil count. As *ITLN1* was reported in a previous Crohn’s disease study^40^, our result suggested an potential relationship between Crohn’s disease and HIV infection^41^.

### 3.3. Integrative Tissue-based Analysis Found Multi-tissue Gene-trait Associations

**Figure 3.**
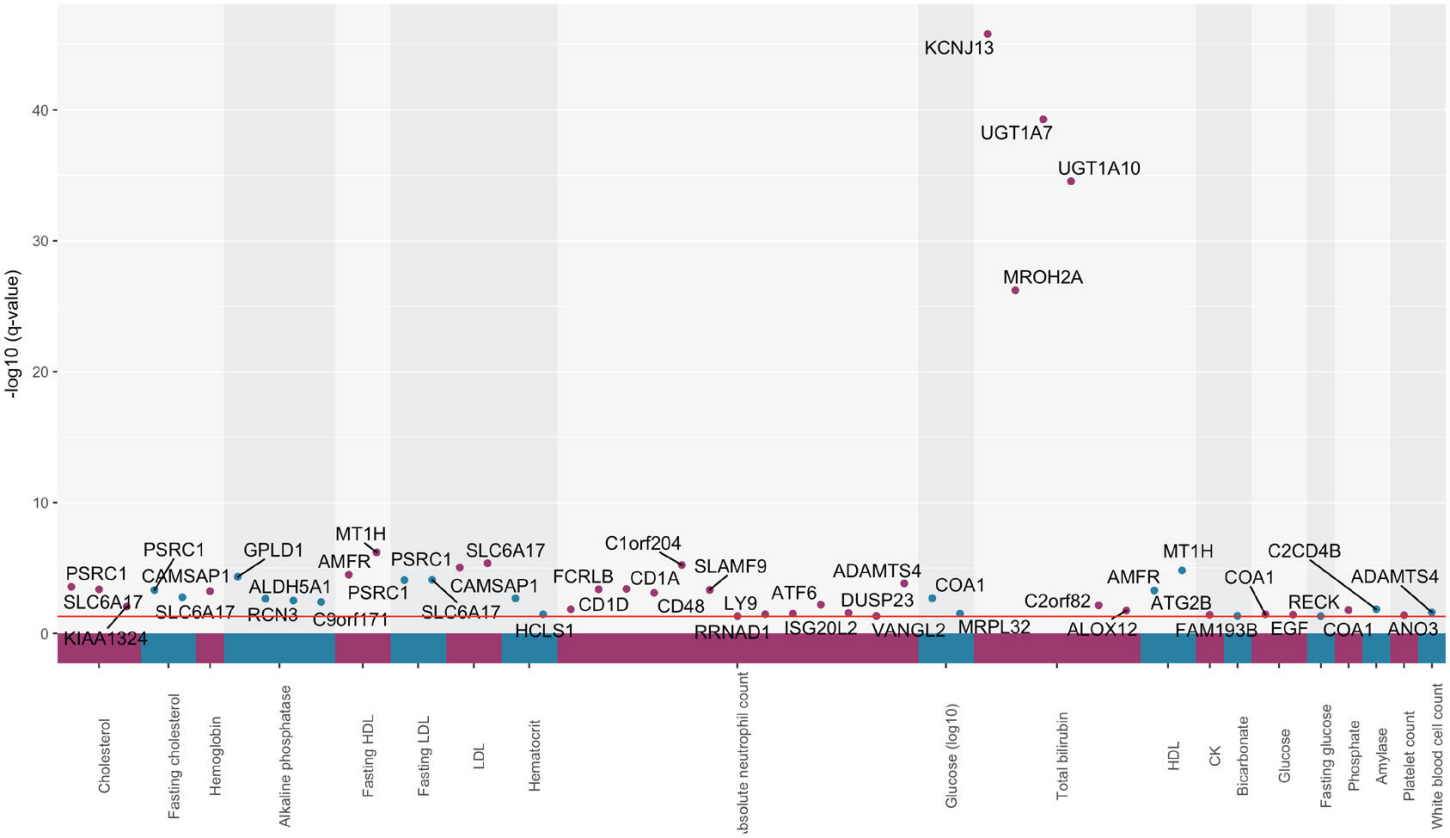
Manhattan plot of gene-trait associations identified by UTMOST. X-axis showed only significant traits. Y-axis was the q-value transformed by -log10. For simplicity, the plot only showed the most significant p-value of a gene-trait pair, which may appear in multiple tissues.

Regardless of tissue, integrative tissue-based analysis using UTMOST identified 50 unique gene-trait pairs (Figure 3). Although it prioritized fewer genes, the integrative tissue-based analysis was more likely to prioritize multiple tissues where genes are expressed. For instance, *PSRC1* is highly expressed in almost all tissues^7^. *PSRC1*-LDL and cholesterol associations were prioritized in at least ten more tissues by integrative tissue-based analysis Most importantly, they were found consistently in the liver which is critically involved in lipid regulation. There was some evidence for distinct associations identified via integrative tissue-based approach (Table 2), such as *ADAMTS4*^42^ with white blood cell count (artery, q-value = 0.023), and *AMFR*^43^ with fasting HDL (most significant in heart, q-value = 3.2e-05).

Other prioritized genes suggested novel associations and potential pleiotropy. Most prioritized genes have been associated with other traits by GWAS according to GWAS Catalog^44^. Similar to the single tissue-based approach, integrative tissue-based analysis prioritized total bilirubin-associated genes from the *UGT1A*^45^ gene locus (*UGT1A7* and *UGT1A10*) across multiple tissues.

## 4. Discussions

This study investigated whether and how TWAS gene prioritization was influenced by tissue context-dependency of eQTLs by comparing two approaches, single tissue-based TWAS (implemented in PrediXcan) and integrative tissue-based TWAS (implemented in UTMOST). PrediXcan evaluated eQTLs’ effects in the context of a single tissue, which did not consider potential multi-tissue effects of eQTLs UTMOST estimated eQTLs’ effect in an integrative tissue setting and increase the chance of identifying multi-tissue eQTLs. We found that both types of analyses could replicate associations discovered by previous studies and identify novel ones. While there were a fair number of overlaps, the two types of analyses prioritized different sets of genes. Single tissue-based analysis identified more unique gene-trait associations. Integrative tissue-based analysis tended to prioritize the same associations in multiple tissues and most importantly association were found in tissues critically related to traits of interest. Results suggest that tissue context-dependency of eQTLs influenced TWAS gene prioritization results.

The comparison raised questions of power and type I error rate of tested TWAS approaches. Integrative tissue context has shown an improved power in identifying eQTLs. As such, integrative tissue-based analysis might have universally greater power in identifying trait-associated genes than single tissue-based analysis. However, in this study, single tissue-based analysis found more validated associations (Table 2). It is hard to tell if integrative tissue-based analysis has universally greater power as expected, whereas single tissue-based analysis happened to identify more false positives. It is also possible that one type of analysis outperformed the other at certain scenarios. A simulation study is necessary to discern these possibilities.

Similar to GWAS, prioritized genes might merely be tag genes for causal ones. Both kinds of analyses prioritized genes at the chromosome 1p13.3 locus where a lipid-related gene, *SORT1*, is located. Single tissue-based analysis associated multiple lipid-related traits with genes that neighbor *SORT1*, such as *SARS*, *CELSR2*, *PSRC1*, and *ALX3*, which all are in the 1p13.3 locus and the same topologically associating domain (TAD^46,47^). Besides *PSRC1*, integrative tissue-based analysis repetitively identified *SLC6A17*. Even though it is not adjacent to *SORT1*, this gene is in the 1p13.3 locus and might serve as a tag gene for causal one(s). Hence, for TWAS, prioritized genes might be merely tag genes and fine-mapping of causal genes may need a larger search boundary than GWAS, such as TADs.

Future investigation or validation experiments may be needed to explain the prioritized genes and/or tissues. For example, *UGT1A1* glucuronidates bilirubin in the liver^48^, but single tissue-based analysis only identified a *UGT1A1*-total bilirubin association in skin. Further analysis found that there was no single *UGT1A1* eQTL identified in liver by either PrediXcan or UTMOST trained on GTEx v6p or v7 data. It is likely that identification of *UGT1A1* eQTLs is limited by tissue sample size (*N*_*liver*_ = 175) or genetic variants may regulate *UGT1A1* via mechanisms other than transcriptional regulation. Another observation of this study was that genes adjacent to *UGT1A1* sporadically showed up as significant in either single tissue-based or integrative tissue-based analysis, including *USP40*, *UGT1A6*, *UGT1A7*, *UGT1A10*, *KCNJ13*, and also *MROH2A*^20^. These genes span 1Mbp in chromosome 2 and locate within the same TAD^46,47^. The repetitive pattern may suggest a specific regulatory activity that targets the whole genetic region of *KCNJ13*-*USP40-UGT1A*-*MROH2A*.

TWAS can prioritize trait-related genes, which may be important for HIV-positive patients regarding genetically informed therapeutic development and drug safety. This study showed that TWAS were able to not only replicate known associations, but also identify novel gene-trait associations. It also suggested the importance of biological context in eQTL studies, and the ensemble of TWAS methods with different transcriptional regulation assumptions gave a more comprehensive picture of gene-trait relationships. In the future, we would like to perform cross-tissue TWAS analysis^12,49^, which aggregate gene-trait association information across all tissues and even across different consortia to further prioritize the trait-related genes and better describe the genetic architecture of complex diseases.

## Figures and Tables

**Table 1.** Significant gene-trait associations (q-value < 0.05) shared by single and integrative tissue-based analysis. The two different analyses shared 11 out of 103 unique significant gene-trait pairs.

Traits	Genes	Methods	#Tissues	Major Tissue Types*
Absolute neutrophil count	*ATF6*	PrediXcan	1	Brain
	*ATF6*	UTMOST	2	Brain, Transformed Fibroblasts
	*Clorf204*	PrediXcan	1	Brain
	*Clorf204*	UTMOST	5	Brain, Ovary, Pituitary
	*VANGL2*	PrediXcan	1	Brain
	*VANGL2*	UTMOST	1	Brain
Alkaline phosphatase	*ALDH5A1*	PrediXcan	9	Artery, Colon, Liver, Lung, Nerve, Pancreas, Skin, Thyroid, Transformed Lymphocytes
	*ALDH5A1*	UTMOST	39	Adipose, Adrenal Gland, Artery, Brain, Breast, Colon, Esophagus, Heart, Liver, Lung, Nerve, Ovary, Pancreas, Pituitary, Prostate, Skeletal Muscle, Skin, Small Intestine, Spleen, Stomach, Test’s, Thyroid, Transformed Lymphocytes, Uterus, Vagina
	*GPLD1*	PrediXcan	2	Artery, Thyroid
	*GPLD1*	UTMOST	24	Adipose, Artery, Brain, Esophagus, Heart, Liver, Lung, Nerve, Pituitary, Prostate, Skeletal Muscle, Skin, Small Intestine, Stomach, Test’s, Thyroid, Transformed Lymphocytes, Vagina, Whole Blood
Cholesterol	*PSRC1*	PrediXcan	9	Brain, Esophagus, Lung, Pancreas, Pituitary, Skeletal Muscle, Skin, Whole Blood
	*PSRC1*	UTMOST	25	Adipose, Brain, Breast, Colon, Esophagus, Heart, Liver, Lung, Nerve, Ovary, Pancreas, Pituitary, Prostate, Skeletal Muscle, Skin, Tests, Uterus, Whole Blood
Fasting cholesterol	*PSRC1*	PrediXcan	9	Brain, Esophagus, Lung, Pancreas, Pituitary, Skeletal Muscle, Skin, Whole Blood
	*PSRC1*	UTMOST	22	Adipose, Brain, Breast, Colon, Esophagus, Heart, Liver, Lung, Nerve, Ovary, Pituitary, Prostate, Skeletal Muscle, Skin, Tests, Uterus, Whole Blood
Fasting LDL	*PSRC1*	PrediXcan	11	Brain, Esophagus, Lung, Pancreas, Pituitary, Skeletal Muscle, Skin, Tests, Thyroid, Whole Blood
	*PSRC1*	UTMOST	27	Adipose, Brain, Breast, Colon, Esophagus, Heart, Liver, Lung, Nerve, Ovary, Pancreas, Pituitary, Prostate, Skeletal Muscle, Skin, Tests, Thyroid, Uterus, Whole Blood
Hemoglobin	*CAMSAP1*	PrediXcan	1	Nerve
	*CAMSAP1*	UTMOST	31	Adipose, Artery, Brain, Breast, Colon, Esophagus, Heart, Liver, Lung, Nerve, Ovary, Prostate, Skeletal Muscle, Skin, Small Intestne, Spleen, Thyroid, Transformed Fibroblasts, Transformed Lymphocytes, Whole Blood
LDL	*PSRC1*	PrediXcan	11	Brain, Esophagus, Lung, Pancreas, Pituitary, Skeletal Muscle, Skin, Tests, Thyroid, Whole Blood
	*PSRC1*	UTMOST	27	Adipose, Brain, Breast, Colon, Esophagus, Heart, Liver, Lung, Nerve, Ovary, Pancreas, Pituitary, Prostate, Skeletal Muscle, Skin, Tests, Thyroid, Uterus, Whole Blood
Total bilirubin	*MROH2A*	PrediXcan	1	Adipose
	*MROH2A*	UTMOST	1	Stomach

* For simplicity, only major tissue types were shown. Skin, heart, esophagus, colon, brain, artery, and adipose have subtypes.

**Table 2.** Validation of some of the TWAS prioritized genes.

GENES	METHODS	TISSUES	Q-VALUE^+^	ACTG TRAITS	GWAS CATALOG REPORTED TRAITS	PMID
*ATF6*	PrediXcan	Brain	1.30E-02	Absolute neutrophil count	White blood cell count	28158719
	UTMOST	Transformed Fibroblasts*, Brain	1.63E-02			
*VANGL2*	PrediXcan	Brain	1.30E-02	Absolute neutrophil count	Multiple sclerosis	24076602
	UTMOST	Brain	4.70E-02			
*ADAMTS4*	UTMOST	Artery	1.50E-04	Absolute neutrophil count*, White blood cell count	Monocyte percentage of white cells	27863252
*ALDH5A1*	PrediXcan	Colon*, Artery, **Liver**, Lung, Nerve, Pancreas, Skin, Thyroid, Transformed Lymphocytes	1.57E-05	Alkaline phosphatase	Liver enzyme levels (alkaline phosphatase)	22001757
	UTMOST	Artery*, Adipose, Adrenal Gland, Brain, Breast, Colon, Esophagus, Heart, **Liver**, Lung, Nerve, Ovary, Pancreas, Pituitary, Prostate, Skeletal Muscle, Skin, Small Intestine, Spleen, Stomach, Testis, Thyroid, Transformed Lymphocytes, Uterus, Vagina	6.58E-03	Alkaline phosphatase		
*ITLN1*	PrediXcan	Stomach	1.04E-05	Alkaline phosphatase, Absolute basophil count, Triglyceride, Viral load	Crohn’s disease	18587394
*CELSR2*	PrediXcan	Brain*, Skeletal Muscle	6.67E-06	Cholesterol, Fasting cholesterol, Fasting LDL, LDL	Total cholesterol, LDL	20686565, 17903299
*PSRC1*	PrediXcan	Lung*, Brain, Esophagus, Pancreas, Pituitary, Skeletal Muscle, Skin, Whole Blood	8.47E-06	LDL*, Cholesterol, Fasting cholesterol, Fasting LDL	Total cholesterol, LDL	20686565, 17903299, 19936222, 17903299, 25101658
	UTMOST	Heart*, Adipose, Brain, Breast, Colon, Esophagus, **Liver**, Lung, Nerve, Ovary, Pancreas, Pituitary, Prostate, Skeletal Muscle, Skin, Testis, Thyroid, Uterus, Whole Blood	1.75E-05			
*CETP*	PrediXcan	Colon	3.24E-17	HDL*, Fasting HDL	HDL cholesterol	25884002, 20686565
*MROH2A*	PrediXcan	Adipose	5.23E-03	Total bilirubin	Bilirubin levels	25884002,21646302
	UTMOST	Stomach	5.97E-27			
*UGT1A1*	PrediXcan	Skin	7.13E-07	Total bilirubin	Bilirubin levels	25884002,21646302
*UGT1A7*	UTMOST	Skin*, Adrenal Gland, Colon, Esophagus, **Liver**, Stomach	5.15E-40	Total bilirubin	Bilirubin levels	25884002,21646302
*APOA1*	PrediXcan	Brain	2.93E-02	Triglyceride	Total cholesterol, Triglyceride, LDL, HDL	20686565,17903299
*APOC3*	PrediXcan	Heart	1.61E-02	Triglyceride	Total cholesterol, Triglyceride, LDL, HDL	20686565,17903299

Bolded tissues are known trait-related tissues.

* denotes the most significant tissue and/or trait that were associated with genes.

+ q-value in the most significant tissue denoted by asterisk.

## References

[1] Mallal S HLA-B*5701 screening for hypersensitivity to abacavir. N. Engl. J. Med 358, 568–579 (2008). 18256392 10.1056/NEJMoa0706135

[2] Rotger M Gilbert syndrome and the development of antiretroviral therapy-associated hyperbilirubinemia. J. Infect. Dis 192, 1381–1386 (2005). 16170755 10.1086/466531

[3] Holzinger ER Genome-wide association study of plasma efavirenz pharmacokinetics in AIDS Clinical Trials Group protocols implicates several CYP2B6 variants. Pharmacogenetics and Genomics 22, 858–867 (2012). 23080225 10.1097/FPC.0b013e32835a450b PMC3614365

[4] Haas DW Pharmacogenetics of efavirenz and central nervous system side effects: an Adult AIDS Clinical Trials Group study. AIDS 18, 2391–2400 (2004). 15622315

[5] Lubomirov R ADME pharmacogenetics: investigation of the pharmacokinetics of the antiretroviral agent lopinavir coformulated with ritonavir. Pharmacogenetics and Genomics 20, 217 (2010). 20139798 10.1097/FPC.0b013e328336eee4

[6] Yuan J Toxicogenomics of nevirapine-associated cutaneous and hepatic adverse events among populations of African, Asian, and European descent. AIDS 25, 1271–1280 (2011). 21505298 10.1097/QAD.0b013e32834779df PMC3387531

[7] Consortium GTEx Genetic effects on gene expression across human tissues. Nature Publishing Group 550, 204–213 (2017). 10.1038/nature24277 PMC5776756 29022597

[8] Li B Evaluation of PrediXcan for prioritizing GWAS associations and predicting gene expression. in 448–459 (WORLD SCIENTIFIC, 2017). doi:10.1142/9789813235533_0041 PMC5749400 29218904

[9] Liu X Functional Architectures of Local and Distal Regulation of Gene Expression in Multiple Human Tissues. American journal of human genetics 100, 605–616 (2017). 28343628 10.1016/j.ajhg.2017.03.002 PMC5384099

[10] Sul JH, Han B, Ye C, Choi T & Eskin E Effectively identifying eQTLs from multiple tissues by combining mixed model and meta-analytic approaches. PLoS Genet 9, e1003491 (2013). 23785294 10.1371/journal.pgen.1003491 PMC3681686

[11] Gamazon ER A gene-based association method for mapping traits using reference transcriptome data. Nat Genet 47, 1091–1098 (2015). 26258848 10.1038/ng.3367 PMC4552594

[12] Hu Y A statistical framework for cross-tissue transcriptome-wide association analysis. bioRxiv 286013 (2018). doi:10.1101/286013 PMC6788740 30804563

[13] Robbins GK Comparison of sequential three-drug regimens as initial therapy for HIV-1 infection. N. Engl. J. Med 349, 2293–2303 (2003). 14668455 10.1056/NEJMoa030264 PMC4767257

[14] Gulick RM Triple-nucleoside regimens versus efavirenz-containing regimens for the initial treatment of HIV-1 infection. N. Engl. J. Med 350, 1850–1861 (2004). 15115831 10.1056/NEJMoa031772

[15] Gulick RM Three- vs four-drug antiretroviral regimens for the initial treatment of HIV-1 infection: a randomized controlled trial. JAMA 296, 769–781 (2006). 16905783 10.1001/jama.296.7.769

[16] Riddler SA Class-sparing regimens for initial treatment of HIV-1 infection. N. Engl. J. Med 358, 2095–2106 (2008). 18480202 10.1056/NEJMoa074609 PMC3885902

[17] Sax PE Abacavir-lamivudine versus tenofovir-emtricitabine for initial HIV-1 therapy. N. Engl. J. Med 361, 2230–2240 (2009). 19952143 10.1056/NEJMoa0906768 PMC2800041

[18] Daar ES Atazanavir Plus Ritonavir or Efavirenz as Part of a 3-Drug Regimen for Initial Treatment of HIV-1: A Randomized Trial. Ann Intern Med 154, 445–456 (2011). 21320923 10.1059/0003-4819-154-7-201104050-00316 PMC3430716

[19] Lennox JL A Phase III Comparative Study of the Efficacy and Tolerability of Three Non-Nucleoside Reverse Transcriptase Inhibitor-Sparing Antiretroviral Regimens for Treatment-Naïve HIV-1-Infected Volunteers: A Randomized, Controlled Trial. Ann Intern Med 161, 461–471 (2014). 25285539 10.7326/M14-1084 PMC4412467

[20] Moore CB Phenome-wide Association Study Relating Pretreatment Laboratory Parameters With Human Genetic Variants in AIDS Clinical Trials Group Protocols. Open Forum Infectious Diseases 2, ofu113–ofu113 (2015). 25884002 10.1093/ofid/ofu113 PMC4396430

[21] Verma A Multiphenotype association study of patients randomized to initiate antiretroviral regimens in AIDS Clinical Trials Group protocol A5202. Pharmacogenetics and Genomics 27, 101–111 (2017). 28099408 10.1097/FPC.0000000000000263 PMC5285297

[22] Turner S Quality control procedures for genome-wide association studies. Curr Protoc Hum Genet Chapter 1, Unit1.19–1.19.18 (2011). 10.1002/0471142905.hg0119s68 PMC3066182 21234875

[23] Purcell S PLINK: a tool set for whole-genome association and population-based linkage analyses. The American Journal of Human Genetics 81, 559–575 (2007). 17701901 10.1086/519795 PMC1950838

[24] Howie BN, Donnelly P & Marchini J A Flexible and Accurate Genotype Imputation Method for the Next Generation of Genome-Wide Association Studies. PLoS Genet 5, e1000529 (2009). 19543373 10.1371/journal.pgen.1000529 PMC2689936

[25] 1000 Genomes Project Consortium A map of human genome variation from population-scale sequencing. Nature Publishing Group 467, 1061–1073 (2010). 10.1038/nature09534 PMC3042601 20981092

[26] Price AL Principal components analysis corrects for stratification in genome-wide association studies. Nat Genet 38, 904–909 (2006). 16862161 10.1038/ng1847

[27] Hall MA PLATO software provides analytic framework for investigating complexity beyond genome-wide association studies. Nature Communications 8, 1167 (2017). 10.1038/s41467-017-00802-2 PMC5660079 29079728

[28] Grady BJ Finding unique filter sets in PLATO: a precursor to efficient interaction analysis in GWAS data. Pac Symp Biocomput 315–326 (2010). doi:10.1142/7628;page:string:Article/Chapter 19908384 PMC2903053

[29] Benjamini Y & Hochberg Y Controlling the False Discovery Rate: A Practical and Powerful Approach to Multiple Testing. Journal of the Royal Statistical Society. Series B (Methodological) 57, 289–300 (1995).

[30] Teslovich TM Biological, clinical and population relevance of 95 loci for blood lipids. Nature 466, 707–713 (2010). 20686565 10.1038/nature09270 PMC3039276

[31] Chasman DI Forty-three loci associated with plasma lipoprotein size, concentration, and cholesterol content in genome-wide analysis. PLoS Genet 5, e1000730 (2009). 19936222 10.1371/journal.pgen.1000730 PMC2777390

[32] Willer CJ Discovery and refinement of loci associated with lipid levels. Nat Genet 45, 1274–1283 (2013). 24097068 10.1038/ng.2797 PMC3838666

[33] Kathiresan S A genome-wide association study for blood lipid phenotypes in the Framingham Heart Study. BMC Med. Genet. 8 Suppl 1, S17 (2007). 17903299 10.1186/1471-2350-8-S1-S17 PMC1995614

[34] Strong A, Patel K & Rader DJ Sortilin and lipoprotein metabolism: making sense out of complexity. Curr. Opin. Lipidol 25, 350–357 (2014). 25101658 10.1097/MOL.0000000000000110 PMC4565516

[35] Chambers JC Genome-wide association study identifies loci influencing concentrations of liver enzymes in plasma. Nat Genet 43, 1131–1138 (2011). 22001757 10.1038/ng.970 PMC3482372

[36] Mero I-L Oligoclonal band status in Scandinavian multiple sclerosis patients is associated with specific genetic risk alleles. PLoS ONE 8, e58352 (2013). 23472185 10.1371/journal.pone.0058352 PMC3589422

[37] International Multiple Sclerosis Genetics Consortium (IMSGC) Analysis of immune-related loci identifies 48 new susceptibility variants for multiple sclerosis. Nat Genet 45, 1353–1360 (2013). 24076602 10.1038/ng.2770 PMC3832895

[38] Weissglas-Volkov D Genomic study in Mexicans identifies a new locus for triglycerides and refines European lipid loci. J. Med. Genet 50, 298–308 (2013). 23505323 10.1136/jmedgenet-2012-101461 PMC3917605

[39] Willer CJ Discovery and refinement of loci associated with lipid levels. Nat Genet 45, 1274–1283 (2013). 24097068 10.1038/ng.2797 PMC3838666

[40] Barrett JC Genome-wide association defines more than 30 distinct susceptibility loci for Crohn’s disease. Nat Genet 40, 955–962 (2008). 18587394 10.1038/NG.175 PMC2574810

[41] Lautenbach E & Lichtenstein GR Human immunodeficiency virus infection and Crohn’s disease: the role of the CD4 cell in inflammatory bowel disease. J. Clin. Gastroenterol 25, 456–459 (1997). 9412950 10.1097/00004836-199709000-00013

[42] Astle WJ The Allelic Landscape of Human Blood Cell Trait Variation and Links to Common Complex Disease. Cell 167, 1415–1429.e19 (2016). 27863252 10.1016/j.cell.2016.10.042 PMC5300907

[43] Benton MC Mapping eQTLs in the Norfolk Island genetic isolate identifies candidate genes for CVD risk traits. American journal of human genetics 93, 1087–1099 (2013). 24314549 10.1016/j.ajhg.2013.11.004 PMC3853002

[44] MacArthur J The new NHGRI-EBI Catalog of published genome-wide association studies (GWAS Catalog). Nucleic Acids Research 45, D896–D901 (2017). 27899670 10.1093/nar/gkw1133 PMC5210590

[45] Bielinski SJ Mayo Genome Consortia: a genotype-phenotype resource for genome-wide association studies with an application to the analysis of circulating bilirubin levels. Mayo Clin. Proc 86, 606–614 (2011). 21646302 10.4065/mcp.2011.0178 PMC3127556

[46] Dixon JR Topological domains in mammalian genomes identified by analysis of chromatin interactions. Nature Publishing Group 485, 376–380 (2012). 10.1038/nature11082 PMC3356448 22495300

[47] Wang Y The 3D Genome Browser: a web-based browser for visualizing 3D genome organization and long-range chromatin interactions. bioRxiv 112268 (2017). doi:10.1101/112268 PMC6172833 30286773

[48] Tukey RH & Strassburg CP Human UDP-glucuronosyltransferases: metabolism, expression, and disease. Annu. Rev. Pharmacol. Toxicol 40, 581–616 (2000). 10836148 10.1146/annurev.pharmtox.40.1.581

[49] Barbeira AN Integrating Predicted Transcriptome From Multiple Tissues Improves Association Detection. bioRxiv 292649 (2018). doi:10.1101/292649 PMC6358100 30668570

[50] Jain D Genome-wide association of white blood cell counts in Hispanic/Latino Americans: the Hispanic Community Health Study/Study of Latinos. Hum. Mol. Genet 26, 1193–1204 (2017). 28158719 10.1093/hmg/ddx024 PMC5968624

